# Analysis of Choroidal Vascularity Index in Keratoconus Patients Using Swept-Source Optical Coherence Tomography-Based Binarization Techniques

**DOI:** 10.1155/2020/1682463

**Published:** 2020-01-08

**Authors:** Rosa Gutierrez-Bonet, Jorge Ruiz-Medrano, Marc Biarnés, Mohammed Abdul Rasheed, Kiran Kumar Vupparaboina, Jay Chhablani, José M. Ruiz-Moreno

**Affiliations:** ^1^Jules Gonin Eye Hospital Fondation Asile des Aveugles, Lausanne, Switzerland; ^2^Puerta de Hierro-Majadahonda University Hospital, Madrid, Spain; ^3^Institut de la Màcula, Barcelona, Spain; ^4^Barcelona Macula Foundation, Barcelona, Spain; ^5^LV Prasad Eye Institute, Kallam Anji Reddy Campus, Hyderabad, India; ^6^Department of Ophthalmology, University of Pittsburgh Medical Center, Pittsburgh, PA, USA; ^7^Castilla-La Mancha University, Albacete, Spain

## Abstract

**Purpose:**

To analyse the vascular density of the choroid in a keratoconus (KC) population using swept-source optical coherence tomography (SS-OCT).

**Methods:**

Prospective, noninterventional study that analysed 97 eyes from 52 KC patients and 145 eyes from 89 healthy controls. The sample was divided in four different age groups. Inclusion criteria were topographic diagnosis of KC using Pentacam, axial length shorter than 26 mm, good quality of the images, and no other systemic or ocular diseases. A 12 mm horizontal single-line SS-OCT b-scan was performed to create a choroidal thickness (CT) profile. Validated automated segmentation and binarization were used in order to analyse choroidal, stromal, and vascular areas.

**Results:**

The percentage of choroidal vascularity (vascular area/total area) was 56.6% in KC patients vs. 49.4% in controls. Aged-adjusted choroidal, stromal, and vascular areas and corrected choroidal percentage of vascularity are statistically increased in KC patients when compared with healthy controls (*p* < 0.001). All these parameters show a decreasing trend with age. Both stromal and vascular areas were thicker in KC patients (*p* < 0.001).

**Conclusions:**

Choroidal, stromal, and vascular areas and corrected choroidal percentage of vascularity are statistically increased in KC patients when compared with healthy controls. All these parameters tend to decrease with age.

## 1. Introduction

The choroid is the vascularised, middle layer of the eye, which can be divided in five histological stratums: Bruch's membrane, choriocapillaris, Haller's and Sattler's layers, and the suprachoidea [1]. The main function of this structure is to nourish the outer retina, showing also thermoregulation capacity, the ability to produce growth factors, and the possibility of modifying its thickness [2].

In recent years, the improvement in retinal imaging devices [3] has allowed the advance of the study of the choroid, and normality patterns of choroidal thickness (CT) profile have been described in children and adults by using enhanced depth imaging or swept-source optical coherence tomography (SS-OCT) [4, 5]. The pathological choroidal changes in certain ophthalmic diseases, such as myopia [6], diabetes [7, 8], aged-related macular degeneration [9], central serous chorioretinopathy [10, 11], polypoidal choroidal vasculopathy [12], and anterior and posterior uveitis [13–16] among others, are well known.

On the other hand, keratoconus (KC) is the most frequent corneal ectasia. The corneal stroma is less rigid and tends to deformation, producing a progressive myopic shift and irregular astigmatism. The main problem is thought to be related to collagen type I fibre degradation by metalloproteinases (MMP) [17]. Latest theories about KC hint to a possible inflammatory component in the pathology of this disease and that might explain the increase in their CT profile compared with healthy population [17–21].

The choroid is a mesh of vessels and stroma in a disorganized pattern, so it is not possible to distinguish its layers as it is normally done with the retina. That is why latest choroidal studies focus on alterations of the CT profile. However, Sonoda et al. described a novel technique that allows the differentiation between the vascular and the stromal components of the choroid, helping the understanding of pathological changes [22].

The purpose of the study is to analyse the vascular density of the choroid in a KC population using SS-OCT and compare it to a healthy population adjusted by age.

## 2. Material and Methods

This was a prospective, cross-sectional, noninterventional study carried out at Jules-Gonin Eye Hospital, University of Lausanne between January 2017 and April 2017. It followed the tenets of the Declaration of Helsinki, and the Essentials of Good Epidemiological Practice issued by Public Health Schweiz, and the Swiss Law and Swiss regulatory authority's requirements. It was approved by the Ethics Committee of the Canton of Vaud, Switzerland (protocol number 2017-00257), and patients signed a specific written consent form prior to the study.

Inclusion criteria were clinical and topographic diagnoses of KC, axial length (AL) minor than 26 mm, good quality scores for both Pentacam (>95% validated data) (Oculus Optikgeräte GmbH, Wetzlar, Germany) and SS-OCT images (allowing a proper binarization), and no systemic diseases. Corneal cross-linking was not considered an exclusion criterion as long as it was performed at least 1 year before taking the images. All those patients suffering from ocular trauma, retinal diseases, glaucoma, or other eye pathology were excluded. All the examinations were performed in the afternoon to prevent diurnal variation of the CT [23].

**Figure 1:**
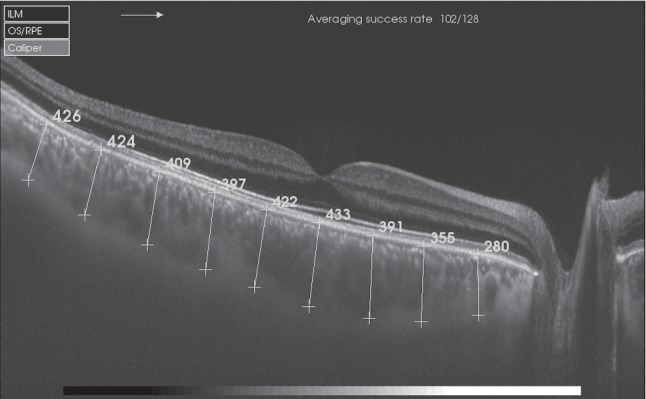
Swept-source optical coherence tomography b-scan of manual choroidal thickness measures in nine locations, from the posterior edge of retinal pigment epithelium to the choroid-sclera junction.

A total of 97 eyes from 52 patients who came to KC follow-up consultation underwent a complete ophthalmic examination, which included best-corrected visual acuity (BCVA), slit-lamp examination, corneal topography before Goldmann applanation tonometry, and fundus examination. Complementary test included Pentacam for corneal topographic examination; IOL Master 500 (Carl Zeiss Meditec, Jena, Germany) for AL calculation in order to exclude those patients who presented an AL longer than 26 mm, given the negative correlation between CT and AL; and Triton SS-OCT (Topcon Co, Japan) to study a 12 mm horizontal single-line b-scan, to generate a CT profile by measuring the distance between the posterior limit of the retinal pigment epithelium and the choroid-sclera junction in 9 different locations (Figure 1) [4, 5, 24]. CT profile was manually determined by two independent investigators (RGB, JRM) in a masked fashion. KC patients were compared with 145 eyes of 89 healthy controls with no ocular or systemic diseases.

**Figure 2:**
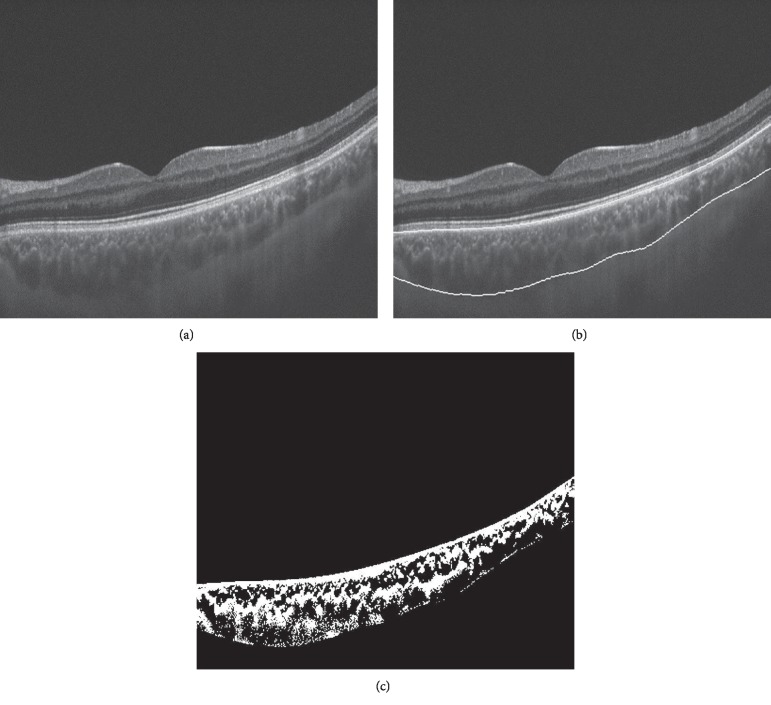
Swept-source optical coherence tomography b-scans along the binarization process of a keratoconus eye. The original B-scan (a) has its inner and outer limits delineated (b). The luminal area and the stromal area are shown in black and white colours, respectively, after the binarization process (c).

Using the same methodology applied by Ruiz-Medrano et al. and described in their paper, total choroidal, stromal, and vascular areas and percentage of vascularity were analysed and quantified. The images of the choroid were automatically binarized using a validated algorithm (Figure 2) [25].

## 3. Statistical Analysis

Univariate analyses were used for descriptive purposes in KC and control groups, using the mean (standard deviation [SD]) and range for quantitative variables, and *n* (percentage) for categorical data. A comparison of demographic and basic clinical features between the two study groups was performed using independent *t*-tests with unequal variances (or Mann–Whitney in nonnormally distributed quantitative variables) and the Fisher exact test for categorical variables. Normality was assessed using graphical approaches.

The following parameters were compared between the two study groups: CT in nine locations (T5, T4, T3, T2, T1, subfoveal, N1, N2, and N3) and four choroidal vascular parameters: choroidal area, stromal region, vascular region, and corrected choroidal percent of vascularity (CCV) understood as vascular region divided by choroidal area × 100. To analyse age-related changes, choroidal vascular parameters were also compared between groups stratified by age in four categories (0 to <20, ≥20 to <40, ≥40 to <60, and ≥60 years old). A test for interaction between age category and study group (KC vs. controls) was conducted for each choroidal vascular parameter. Choroidal vascular parameters and age were also compared across KC severity according to the Pentacam HR software classification system (Early KC: <2; Moderate KC: 2 to <3; and Advanced KC: ≥3) using ANOVA or Kruskal–Wallis tests. To account for the intereye correlation in bilateral patients, we selected a hierarchal multivariate linear regression model with generalized estimating equations (GEE) [26] and robust standard errors using an independent working correlation [27]. These models were adjusted by age.

Agreement in CT measures between observers in eyes with KC was evaluated graphically using Bland–Altman plots [28] and quantitatively using the coefficient of repeatability (CR, which provides an interval within which 95% of test-retest measurement differences lie) [29].

No adjustments were made for multiple comparisons [30]. All analyses were conducted using Stata IC 13.1 (StataCorp LP, College Station; TX). A two-tailed *p* value of <0.05 was considered statistically significant.

## 4. Results

The study included a total of 242 eyes, 97 eyes from 52 KC patients and 145 eyes of 89 healthy controls. Their demographic and clinical characteristics by group are shown in Table 1. The topographic features of patients with KC are shown in Table 2.

Table 3 shows the intergroup comparison of CT in 9 locations along the horizontal meridian and the choroidal vascular coefficients adjusted by age. KC patients had consistently greater values than controls in all choroidal parameters.

Choroidal area, stromal region, vascular region, and corrected choroidal percentage of vascularity between groups were compared by age categories (Table 4). There was a progressive descending trend with age in the point estimate for all choroidal parameters, suggesting that choroidal vascular parameters tend to decrease with aging. This hypothesis (that there are changes in vascular parameters by groups with progressive older age) was tested formally with a *p* value for interaction, which was highly statistically significant for all four variables (*p* < 0.0001 each).

**Figure 3:**
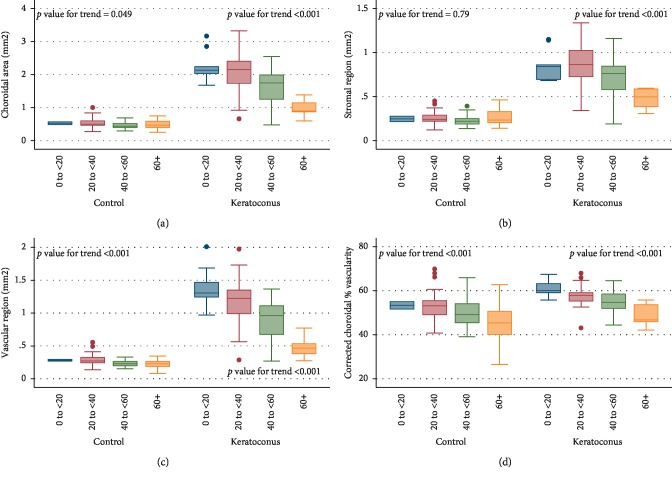
Box plot results of each choroidal parameter by age category: (a) choroidal area; (b) stromal region; (c) vascular region; (d) corrected choroidal percent of vascularity. Represented *p* values are for trend for age category for each group separately. Eyes with keratoconus have greater values than controls and generally follow a more marked descending trend with age.

Figure 3 shows the corresponding box plots results. *p* Values for trend in all choroidal parameters (Choroidal area, Stromal region, Vascular region, and Corrected choroidal percentage of vascularity) were statistically significant for KC (all *p* values <0.001), which means that they decreased progressively with increasing age category. In controls, Choroidal area showed a borderline trend toward a decrease with age category (*p*=0.049); there was no clear trend for Stromal region (*p* value = 0.79), while Vascular region and Corrected choroidal percentage of vascularity decreased clearly with greater age category (*p* value <0.001).

The choroidal vascular parameters by topographic stage of KC are shown in Table 5. There were no statistically significant differences in any choroidal parameter between KC stages (*p* ≥ 0.36). Age did not differ either across KC categories (*p*=0.76).

Table 6 shows four GEE models of the effect of KC on each of the four choroidal vascular parameters adjusted for age. The models confirm that having KC increases all choroidal vascular parameters, from 0.52 mm^2^ for the Stromal region to 1.30 mm^2^ for the Choroidal area in a horizontal foveal B-scan; this effect was independent of age. For example, a 44-year-old KC patient is predicted to have a mean choroidal area of approximately 1.85 mm^2^ as compared with 0.55 mm^2^ for a 44-year-old control, more than a threefold increase.

**Figure 4:**
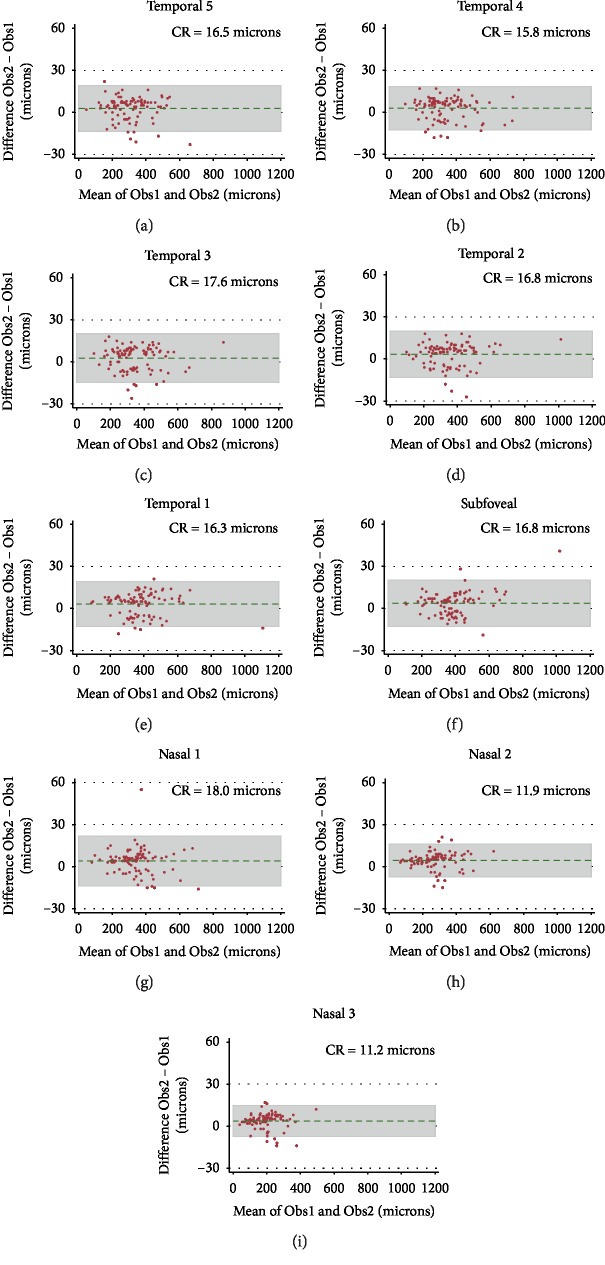
Bland–Altman plots and corresponding CR for interobserver agreement in choroidal thickness measurements in each of the 9 positions: (a, c) locations T5, T4, and T3, respectively; (d, e) locations T2 and T1 and (f) subfoveal, respectively; (g, i) locations N1, N2, and N3, respectively. CR: coefficient of repeatability; N: nasal; T: temporal.

Figure 4 shows Bland–Altman plots and coefficients of repeatability to test the agreement between observers in CT in the 9 locations. As expected, better agreement was found for thinner choroids.

## 5. Discussion

The origin of KC remains unknown. Corneal collagen degradation produces a disruption at the level of the epithelial basal membrane and more importantly, stromal thinning. Collagen type I, the main component of the corneal stroma, is not correctly organized, which leads to a decrease in tensile force and, consequently, a tendency to deformation [31]. It is understood as a noninflammatory ectasia; however, recent studies have shown increased levels of proinflammatory mediators [17]. Traces of these molecules have been reported in the corneal epithelium and in the tear film, being responsible for extracellular matrix degradation and stromal thinning caused by the activation of MMPs [17, 19].

Changes in CT profile in KC patients have already been described [21]. However, the exact changes taking place in the choroid itself are still unclear. The use of choroidal binarization techniques may shed some light regarding the structural changes taking place in the choroid of patients suffering from KC, which may potentially induce an increase in CT.

In the present study, a statistically significant difference in CT profile was found between KC patients and healthy controls in all nine measured locations (Table 3), and these effects are independent of age (Table 6). Choroidal area, stromal, and vascular region and corrected choroidal percentage of vascularity are also statistically increased in KC patients when compared with healthy controls (*p* < 0.001, Table 3). In the same way as in other studies, all these parameters show a decreasing trend with age in both KC and healthy controls.

Both stromal and vascular area are thicker in KC (*p* < 0.001), but there is a larger increase in vascular area compared with stromal area in KC vs. healthy controls, which involves an increase in the corrected choroidal vascularity index of 7.19% (*p* < 0.001) when compared with healthy controls. These results may point toward vascular dilation and inflammatory stromal infiltration in KC patients contributing to the increase in CT, with vascular dilation as the major component of choroidal thickening. Histological studies about some inflammatory diseases, such as Vogt–Koyanagi–Harada (VKH), have described inflammatory cells at stromal level with an increase in CT during inflammatory phase, which returns to normality after cortisone treatment [13, 14, 32–34]. These inflammatory cells activate the production of proinflammatory cytokines, the same that can be found at the cornea or tear film of KC patients. The cytokines may activate endothelial nitric oxide synthase, producing choroidal vascular dilation [35, 36]. In this context, a study by Shetty et al. [17] showed that cyclosporine drops were able to arrest disease progression by reducing MMP-9 tear and corneal levels.

On the other hand, Sobrino and coworkers [37] have recently proved overexpression of Toll-like receptors 2 (TLR2) and 4 (TLR4) in monocytes and neutrophils of KC patients and higher serum levels of interleukin-1B (IL-1B), IL-6, tumour necrosis factor-*α*, MMP-9, the main inflammatory mediators found at corneal epithelium and tear film. These results suggest that there may exist an imbalance of proinflammatory and anti-inflammatory factors, and an increase in oxidative stress not only at cornea level but also systemically.

There were no significant differences between the different KC stages. Nevertheless, KC patients and healthy controls show a tendency towards CT thinning with age. This thinning is statistically significant in KC (all *p* values <0.001), and the results demonstrate choroidal thinning mainly due to loss of vascular area. The ectasia typically reaches stabilization around the third-fourth decade of life and at the same time, CT and choroidal area diminish. This relationship might be justified by the decrease in proinflammatory mediators, which could lead to conus stabilization. In the same way, decreased levels of nitric oxide could explain why vascular areas get thinner around this period.

The results of this study are similar to those found by Ruiz-Medrano et al. [25] when they analysed the evolution of choroidal vascular density with age in a healthy population. They found a statistically significant difference in the percentage of vascular/total area, which decreases with age, while stromal area remains stable. In our study, KC patients showed the same tendency, with the stromal component of the choroid displaying more stability than the vascular area. Healthy controls showed the same trend as Ruiz-Medrano et al. as well, even though our sample was smaller. This variability at stromal level in KC patients could support the inflammatory infiltration hypothesis.

Our study has several limitations. Both CT and the analysis of the different choroidal areas were studied using a single-line, fovea-centered scan protocol, so isolated alterations in the macular CT profile may have gone unnoticed. The choroid is a disorganized vascular network, so differently oriented scans could be necessary to confirm the results. There may also be concerns about quantification of dark and white regions as vessels and stroma, respectively; nevertheless, this theory is commonly accepted. The hypothesis of high astigmatism distorting the images of the choroid was discarded given that retinal thickness was unaltered in the sample.

In conclusion, CT, choroidal area, stromal, and vascular regions and corrected choroidal percentage of vascularity are statistically increased in KC patients when compared with healthy controls. All these parameters showed a statistically significant decreasing trend with age, and our results explain that this choroidal thinning is mainly due to the loss of vascular area. Further studies will be necessary in order to confirm these results.

## Figures and Tables

**Table 1:** Patients' baseline characteristics.

	KC (97 eyes/52 patients)	Control (145 eyes/89 patients)	*p* value
Sex (female)	19 (36.5%)	40 (44.9%)	0.38
Age, years	35.0 (13.7)	52.7 (17.4)	<0.0001
Eye (right)	48 (49.5%)	68 (46.9%)	0.70
BCVA, Snellen	20/24^*∗*^	20/21	<0.0001
KC severity^†^			
Early	37 (38.1%)	NA	—
Moderate	33 (34.0%)		
Advanced	27 (27.8%)		
Cross-linking	24 (24.7%)	NA	—
Other surgeries^‡^			
None	91 (93.8%)	NA	—
PK	5 (5.2%)		
DALK	1 (1.0%)		

Baseline features of participants by study group. Categorical variables expressed as *n* (percentage) and quantitative variables as means (standard deviation). Percentages may not add up to 100% due to rounding. Sex and age are reported at patient level; all other variables are reported at eye level. BCVA: best-corrected visual acuity; KC: keratoconus; NA: does not apply; PK: penetrating keratoplasty. ^*∗*^*n* = 94 eyes. ^†^Based on Pentacam HR® software classification system. ^‡^Patients had surgery after the current study.

**Table 2:** Topographic characteristics of keratoconus patients.

Age group (*n*)	TKC	Minimum pachymetry	Posterior elevation	*K* mean	*K* max	Axial length
0 to <20 (10)	1.8 (1.3)	484.7 (39.0)	40.8 (32.6)	47.29 (3.61)	54.94 (8.53)	23.77 (1.03)
20 to <40 (59)	2.0 (1.0)	447.9 (69.3)	50.7 (27.8)	48.02 (5.23)	54.39 (6.61)	23.81 (1.16)
40 to <60 (23)	2.0 (1.0)	476.1 (58.8)	46.5 (30.2)	47.66 (5.77)	53.89 (8.31)	24.49 (1.06)
≥60 (5)	2.1 (1.7)	490.8 (138.5)	79.8 (68.1)	52.28 (9.04)	61.68 (18.28)	25.47 (0.42)
Overall (97)	2.0 (1.0)	460.3 (68.8)	50.2 (32.1)	48.08 (5.46)	54.71 (8.11)	24.06 (1.17)

Topographic characteristics of patients with keratoconus by age group (in years). Measures represent mean (standard deviation). Minimum pachymetry and posterior elevation in microns, *K* mean and *K* max in diopters; axial length in millimeters. TKC: topographic keratoconus classification.

**Table 3:** Choroidal thickness and vascular coefficients in both groups.

		KC	Control	KC-control (95% CI)	*p* value
Temporal 5 (*μ*m)		305.8	227.7	+78.1 (53.0 to 103.3)	<0.0001
Temporal 4 (*μ*m)		334.9	243.6	+91.3 (63.5 to 119.1)	<0.0001
Temporal 3 (*μ*m)		344.5	254.6	+89.9 (62.1 to 117.6)	<0.0001
Temporal 2 (*μ*m)		354.4	269.1	+85.2 (55.8 to 114.7)	<0.0001
Temporal 1 (*μ*m)		378.2	285.3	+92.9 (62.5 to 123.3)	<0.0001
Subfoveal (*μ*m)		385.0	290.7	+94.3 (64.1 to 124.6)	<0.0001
Nasal 1 (*μ*m)		338.8	259.3	+79.5 (52.7 to 106.3)	<0.0001
Nasal 2 (*μ*m)		270.0	203.4	+66.6 (42.1 to 91.1)	<0.0001
Nasal 3 (*μ*m)		192.3	145.4	+46.8 (27.9 to 65.8)	<0.0001
Choroidal area (mm^2^)		1.92	0.50	+1.42 (1.29 to 1.53)	<0.0001
Stromal region (mm^2^)		0.80	0.25	+0.55 (0.50 to 0.60)	<0.0001
Vascular region (mm^2^)		1.10	0.25	+0.85 (0.78 to 0.92)	<0.0001
CCV (%)		56.60	49.40	+7.19 (5.66 to 8.73)	<0.0001

Comparison of choroidal thickness and vascular coefficients between keratoconic patients and healthy controls. Results represent mean (standard deviation) except when indicated. CCV: corrected choroidal percent vascularity; KC: keratoconus.

**Table 4:** Vascular coefficients by age group.

		KC	Control	KC-control (95% CI)	*p* value
1^st^ tertile, from 14 to 35 years (*n* = 92, KC: 60, control: 32)	CA (mm^2^)	2.11	0.57	+1.55 (1.41 to 1.68)	<0.0001
	SR (mm^2^)	0.86	0.26	+0.60 (0.54 to 0.66)	<0.0001
	VR (mm^2^)	1.23	0.31	+0.93 (0.84 to 1.01)	<0.0001
	CCV (%)	58.36	54.78	+3.58 (1.17 to 5.99)	0.004
2^nd^ tertile, from 36 to 53 years (*n* = 72, KC: 32, control: 40)	CA (mm^2^)	1.69	0.50	+1.19 (1.00 to 1.38)	<0.0001
	SR (mm^2^)	0.74	0.25	+0.49 (0.41 to 0.57)	<0.0001
	VR (mm^2^)	0.93	0.25	+0.68 (0.57 to 0.79)	<0.0001
	CCV (%)	54.51	49.50	+5.01 (2.70 to 7.32)	0.0001
3^rd^ tertile, from 54 to 85 years (*n* = 78, KC: 5, control: 73)	CA (mm^2^)	0.98	0.47	+0.51 (0.14 to 0.88)	0.02
	SR (mm^2^)	0.48	0.25	+0.23 (0.07 to 0.38)	0.02
	VR (mm^2^)	0.49	0.22	+0.27 (0.03 to 0.50)	0.03
	CCV (%)	48.83	46.99	+1.84 (-5.20 to 8.87)	0.53

Comparison of vascular coefficients between keratoconic patients and healthy controls by tertiles of age (in years). The groups do not contain exactly the same number of subjects because there were some cases with exactly the same value for age used to separate categories. Results represent mean (standard deviation) except when indicated. CA: choroidal area; CCV: corrected choroidal percent of vascularity; KC: keratoconus; SR: stromal region; VR: vascular region.

**Table 5:** Vascular coefficients in different keratoconus stages.

	Early KC (*n* = 37)	Moderate KC (*n* = 33)	Advanced KC (*n* = 27)	*p* value
Age, years	34.1 (13.7)	33.5 (12.7)	36.0 (14.0)	0.76
Choroidal area (mm^2^)	1.95 (0.57)	1.81 (0.62)	2.00 (0.53)	0.39
Stromal region (mm^2^)	0.80 (0.25)	0.76 (0.24)	0.84 (0.21)	0.45
Vascular region (mm^2^)	1.13 (0.34)	1.02 (0.40)	1.14 (0.33)	0.36
CCV (%)	57.62 (4.55)	55.43 (5.87)	56.63 (3.75)	0.43

Comparison of vascular coefficients across topographic KC stage as defined by the Pentacam HR software classification system (early KC: <2; moderate KC: 2 to <3; and advanced KC: ≥3) and age. Results represent mean (standard deviation). CCV: corrected choroidal percent of vascularity; KC: keratoconus.

**Table 6:** Effect of having a keratoconus as compared with being a control on each choroidal vascular parameter adjusted for age.

	Coefficient (95% CI)	Standard error	*p* value
Choroidal area			
Keratoconus	1.30 (1.15 to 1.45)	0.08	<0.001
Age, years	−0.007 (−0.011 to −0.004)	0.002	<0.001
Intercept	0.55 (0.51 to 0.59)	0.021	<0.001
Stromal region			
Keratoconus	0.52 (0.46 to 0.58)	0.03	<0.001
Age, years	−0.002 (−0.003 to −0.000)	0.001	0.03
Intercept	0.26 (0.25 to 0.28)	0.01	<0.001
Vascular region			
Keratoconus	0.76 (0.67 to 0.85)	0.05	<0.001
Age, years	−0.005 (−0.008 to −0.003)	0.001	<0.001
Intercept	0.28 (0.26 to 0.31)	0.01	<0.001
CCV			
Keratoconus	4.07 (2.22 to 5.93)	0.95	<0.001
Age, years	−0.19 (−0.25 to −0.13)	0.03	<0.001
Intercept	50.66 (49.36 to 51.95)	0.66	<0.001

Effect of having a keratoconus as compared with being a control on each choroidal vascular parameter, adjusted for age (mean-centered at 44.4 years old), using generalized estimating equations with robust standard errors to account for intereye correlation. CCV: corrected choroidal percent of vascularity.

## Data Availability

The data used to support the findings of this study are available from the corresponding author upon request.
